# Indirect Calorimetry Overestimates Oxygen Consumption in Young Children: Caution is Advised Using Direct Fick Method as a Reference Method in Cardiac Output Comparison Studies

**DOI:** 10.1007/s00246-019-02238-5

**Published:** 2019-11-18

**Authors:** Theodor S. Sigurdsson, Lars Lindberg

**Affiliations:** 1 Department of Pediatric Anesthesia and Intensive Care, Children’s Hospital, Skåne University Hospital, Lund, Sweden; 2 Department of Anesthesia and Intensive Care, Landspítalinn University Hospital, Reykjavík, Iceland

**Keywords:** Cardiac output, Cardiac surgery, Children, Indirect calorimetry, Oxygen consumption, Reverse fick method

## Abstract

Direct Fick method is considered a standard reference method for estimation of cardiac output. It relies on indirect calorimetry to measure oxygen consumption. This is important as only a minor measurement error in oxygen consumption can result in false estimation of cardiac output. A number of studies have shown that indirect calorimetry overestimates oxygen consumption in adults. The aim of this prospective single center observational method comparison study was to compare the determination of oxygen consumption by indirect calorimetry and reverse Fick method in pediatric patients. Forty-two children mean age 352 days (range 30 to 1303 days) and mean weight 7.1 kg (range 2.7–13.6 kg) undergoing corrective cardiac surgery were included in the study. The mean (standard deviation) oxygen consumption by reverse Fick method was 43.5 (16.2) ml/min and by indirect calorimetry 49.9 (18.8) ml/min (*p* < 0.001). Indirect calorimetry overestimated the reverse Fick oxygen consumption by 14.7%. Bias between methods was 6.5 (11.3) ml/min, limits of agreement (LOA) − 15.7 and 28.7 ml/min and percentage error of 47.7%. A significant bias and large percentage error indicates that the methods are not interchangeable. Indirect calorimetry and the direct Fick method should be used with caution as a reference method in cardiac output comparison studies in young children.

**Electronic supplementary material:**

The online version of this article (10.1007/s00246-019-02238-5) contains supplementary material, which is available to authorized users.

## Introduction

More than a century ago the German physician Adolf Eugen Fick (1829–1901) hypothesized that it should be possible to estimate cardiac output if the arterio-mixed venous oxygen content difference and systemic oxygen consumption are known [1, 2]. Time and progress in medical science would prove Fick right, as the 1956 Nobel prize was awarded to the pioneers in cardiac catheterization who confirmed the Fick principle in humans [3, 4].

The so-called direct Fick method has since then been considered a standard reference method for the estimation of cardiac output [5, 6]. Direct Fick method determines cardiac output by dividing the oxygen consumption (${\text{mV}}_{{{\text{O}}_{2} }}$), with the difference between arterial (${\text{Ca}}_{{{\text{O}}_{2} }}$) and mixed venous oxygen content (${\text{Cv}}_{{{\text{O}}_{2} }}$) (Eq. 1).

1 $$Q = {\text{mV}}_{{{\text{O}}_{2} }} /\left( {{\text{Ca}}_{{{\text{O}}_{2} }} - {\text{Cv}}_{{{\text{O}}_{2} }} } \right).$$

Oxygen consumption (${\text{mV}}_{{{\text{O}}_{2} }}$) is measured with indirect calorimetry, but in recent years with increasing demand on precision and accuracy in comparison studies, there has been a growing concern regarding its technical limitations and possible errors.

In the reverse Fick method, oxygen consumption (${\text{cV}}_{{{\text{O}}_{2} }}$) can be calculated if cardiac output (*Q*) has been measured by some other means and both arterial (${\text{Ca}}_{{{\text{O}}_{2} }}$) and mixed venous blood gas (${\text{Cv}}_{{{\text{O}}_{2} }}$) values are known.

2 $${\text{cV}}_{{{\text{O}}_{2} }} = Q\;*\;\left( {{\text{Ca}}_{{{\text{O}}_{2} }} - {\text{Cv}}_{{{\text{O}}_{2} }} } \right).$$

A number of studies have shown discrepancies between measured ${\text{mV}}_{{{\text{O}}_{2} }}$ by indirect calorimetry and calculated ${\text{cV}}_{{{\text{O}}_{2} }}$ by reverse Fick method in adults [7–10]. As the direct Fick method relies on measured oxygen consumption by indirect calorimetry, measurement errors could cause false estimations of cardiac output.

The hypothesis of the study is that there is not a significant difference in the estimation of oxygen consumption using indirect calorimetry versus the reverse Fick method. The aim of this study was to investigate if oxygen consumption was overestimated in a cohort of young children undergoing corrective cardiac surgery.

## Methods

### Patient and Data Collection

Forty-two patients undergoing congenital cardiac surgery were included in this study which was a part of a larger ongoing single center prospective method comparison study [11]. The study was approved and registered by the Ethics Committee of Lund University, Sweden (Dnr 2,013,636). Inclusion criteria included parental consent, elective open heart surgery, weight less than 15 kg, pulse oximetry saturation > 93%, inspired oxygen fraction (${\text{Fi}}_{{{\text{O}}_{2} }}$) 0.21–0.40, endotracheal intubation with a cuffed tube without leakage, tidal volumes of 7–8 ml/kg, sinus rhythm and echocardiography not showing signs of a significant intracardiac shunt after coming off the heart–lung bypass machine. Exclusion criteria included arrythmias and active bleeding after surgery.

### Oxygen Consumption Measured by Indirect Calorimetry (${\text{mV}}_{{{\text{O}}_{2} }}$)

Measured oxygen consumption (${\text{mV}}_{{{\text{O}}_{2} }}$) was obtained by indirect calorimetry using GE Healthcare Datex-Ohmeda S/5 Compact Anaesthesia Monitor (Datex Ohmeda, Inc., Madison, WI, USA) with a E-CAiOVX module, specific Pedilite + paediatric flow sensor and adjusted to paediatric mode [12, 13]. Measurements can be collected after a 5-min warm-up period, including an automatic calibration, after which the monitor can yield a continuous $V_{{{\text{O}}_{2} }}$ reading in 5-min intervals. The S/5 monitor was calibrated annually by biomedical engineering staff against a Datex-Ohmeda calibration syringe (containing specific proportion of gases) as recommended by the manufacturer [14].

### Oxygen Consumption Calculated by Reverse Fick Method (${\text{cV}}_{{{\text{O}}_{2} }}$)

Calculated oxygen consumption (${\text{cV}}_{{{\text{O}}_{2} }}$) was calculated using the reverse Fick method according to Eq. 2.

### Arterial and Mixed Venous Oxygen Content ${\text{(Ca}}_{{{\text{O}}_{2} }} ,\;{\text{Cv}}_{{{\text{O}}_{2} }} )$

Blood gases were taken simultaneously. Arterial blood gas was taken from the radial artery. Mixed venous blood gas was taken from the pulmonary truncus by direct puncture by the surgeon. Blood gas analysis was done with the ABL800 Flex Radiometer (Radiometer AS, Brønshøj, Denmark).

Arterial oxygen content (${\text{Ca}}_{{{\text{O}}_{2} }}$) was calculated as:

3 $${\text{Ca}}_{{{\text{O}}_{2} }} = {\text{Sa}}_{{{\text{O}}_{2} }} \times {\text{Hba}} \times 1.39 + 0.0031 \times {\text{Pa}}_{{{\text{O}}_{2} }} .$$

${\text{Sa}}_{{{\text{O}}_{2} }}$ is arterial oxygen saturation, Hba is arterial hemoglobin, ${\text{Pa}}_{{{\text{O}}_{2} }}$ is arterial partial pressure of oxygen.

Mixed venous oxygen content (${\text{Cv}}_{{{\text{O}}_{2} }}$) was calculated as:

4 $${\text{Cv}}_{{{\text{O}}_{2} }} = {\text{Smv}}_{{{\text{O}}_{2} }} \times {\text{Hbv}} \times 1.39 + 0.0031 \times {\text{Pmv}}_{{{\text{O}}_{2} }} .$$

${\text{Smv}}_{{{\text{O}}_{2} }}$ is mixed venous oxygen saturation, Hbv is venous hemoglobin, ${\text{Pmv}}_{{{\text{O}}_{2} }}$ is mixed venous partial pressure of oxygen.

### Cardiac Output Measurement

Cardiac output (*Q*) was obtained by the COstatus monitor device (Transonic Systems, Inc., Ithaca, NY, USA) which uses a specific single-use extracorporeal arteriovenous circuit (AV loop) connected to arterial and central venous catheters already in place and external ultrasound senors (attaced to the AV loop) to detect transcardiopulmonary blood dilution [15]. This dilution is estimated by the change in ultrasound velocity of blood after the transcardiopulmonary passage. The ultrasound velocity of blood is defined in a certain range (1.560–1.585 m/s) as there are fluctuations in total blood protein concentration (sum of proteins in the plasma and red cells) that influence velocity. As a small bolus (0.5–1.0 ml/kg) of normothermic saline (which has an ultrasound velocity of 1.530 m/s) is injected into the venous side of the AV loop, there will be a transient decrease in ultrasound velocity of the blood. This decrease is detected by an ultrasound sensor on the arterial side of AV loop. The COstatus device, creates a dilution curve based on this information, that is then displayed on a specific monitor and used to estimate the cardiac output using the Stewart–Hamilton indicator dilution principle [16–18]. The COstatus monitor device is considered minimally invasive as it only relies on available central venous and arterial catheters and has been shown to be accurate, precise and safe to use in number of pediatric studies [19–21]. Annual calibration was done by the manifacturer for the duration of the study. The device automatically performs a baseline determination of the ultrasound blood velocity in the AV loop before each measurement session.

### Experimental Protocol

Anesthesia was achieved in all patients in the same manner, fentanyl (5 mcg/kg) and penthothal (5 mg/kg) for induction, pancuronium (0.2 mg/kg) to facilitate endotracheal intubation with a cuffed tube and isoflurane (0.5–1.0%) for maintainance. Stable ventilation was maintained with the Dräger Apollo anesthesia machine (Drägerwerk AG and Co., Lübeck, Germany) using tidal volumes of 7–8 ml/kg and ${\text{Fi}}_{{{\text{O}}_{2} }} < 0.{4}0.$ It was ensured that no leakage was present in the breathing circuit, by carefully monitoring that inspiratory and expiratory tidal volumes and that the volume curve during measurements. All children received peripheral arterial catheters in the radial artery (Neoflow 24 G < 5 kg patient and Venflow 22 G > 5 kg patient, BD Ltd., Wokingham, UK) and central venous catheters (Multicath triple lumen 6 cm, 4.5 F, Vygon Ltd., Swindon, UK) in the right internal jugular vein. The COstatus monitor was then connected to arterial- and central venous catheters in preparation for cardiac output measurements. After the surgical correction, a postoperative transesophageal echocardiography confirmed a biventricular circulation without shunts. The measurement session consisted of five consecutive cardiac output measurements with a simultaneous measurement of ${\text{mV}}_{{{\text{O}}_{2} }}$ by indirect calorimetry by S/5 Compact Anaesthesia Monitor. Blood gases were taken immediately after the last cardiac output measurement. ${\text{cV}}_{{{\text{O}}_{2} }}$ was then calculated using reverse Fick method as described previously.

### Statistical Analysis

Data was registered in Windows Excel (Microsoft Corporation, Redmond, WA, USA) and the statistical analysis was done with Statistica version 13 (Dell, Inc., Tulsa, OK, USA).

All data is expressed as the mean (standard deviation) in the paper unless indicated otherwise.

A priory statistical power analysis was performed for sample size estimation based on data from an earlier study comparing oxygen consumption in adults [8]. In that study, the bias was 19 ml/min and the SD 20 ml/min resulting in an effect size of 0.95. Using the G-Power 3.1.9.2 software, with paired *t* test for difference of means, an *α* error value of 0.05 and power value of 0.90, it was estimated that a sample size of at least 11 subjects were needed. However, as we had available data from an ongoing cardiac output comparison study, we could include 42 patients in this study.

The differences between the methods were analyzed using the Student’s *t* test for paired samples.

Bland–Altman analysis was used to estimate the mean difference (bias) between ${\text{cV}}_{{{\text{O}}_{2} }}$ and ${\text{mV}}_{{{\text{O}}_{2} }}$ and plotted against the average of the comparison $\left( {{\text{cV}}_{{{\text{O}}_{2} }} + {\text{mV}}_{{{\text{O}}_{2} }} } \right)/2.$ The 95% LOA were calculated as mean bias ± 1.96 * SD (standard deviation of the bias). LOA analysis was performed to determine if the two methods agreed sufficiently to be interchangeble. The Shapiro–Wilks test was used to confirm normal distribution of the difference between different methods. The percentage error was calculated according to Critchley and Critchley as 1.96 × SD of the bias/mean $V_{{{\text{O}}_{2} }}$ of both methods × 100% [22].

$${\text{PE}} = \frac{{1.96\;*\;{\text{SDbias}}}}{{V_{{{\text{O}}_{2} }} {\text{mean}}}}\;*\; \left( {100\% } \right).$$

A percentage error of < 30% for comparisons was defined as the criterion for interchangeability. A *p* value of < 0.05 was regarded as statistically significant.

## Results

This study involved 42 children with a mean age of 352 (357) days (range 30 to 1303 days), a mean weight of 7.1 (3.0) kg (range 2.7 to 13.6 kg) and a mean body surface area (BSA) of 0.3 (0.11) m^2^ (range 0.10 to 0.50 m^2^).

The mean cardiac output values from 5 consecutive measurements were calculated from a total of 210 cardiac output measurements in 42 children and converted to ${\text{cV}}_{{{\text{O}}_{2} }}$ using the reverse Fick method. Indirect calorimetry measurements were simultaneously done in the same 42 children. Oxygen consumption values for both methods are presented in Table 1.

**Table 1:** Simultaneously calculated oxygen consumption by reverse Fick and measured by indirect calorimetry in 42 children after surgical correction

Patient	Oxygen consumption		Patient	Oxygen consumption	
	${\text{cV}}_{{{\text{O}}_{2} }}$ (ml/min)	${\text{mV}}_{{{\text{O}}_{2} }}$ (ml/min)		${\text{cV}}_{{{\text{O}}_{2} }}$ (ml/min)	${\text{mV}}_{{{\text{O}}_{2} }}$ (ml/min)
1	44	31	22	52	80
2	23	19	23	46	47
3	40	27	24	30	27
4	60	67	25	33	35
5	33	52	26	63	62
6	19	25	27	67	90
7	67	83	28	22	36
8	33	28	29	32	54
9	50	41	30	24	31
10	33	56	31	56	64
11	43	36	32	34	54
12	52	61	33	70	80
13	40	42	34	62	60
14	92	69	35	31	37
15	26	23	36	37	41
16	62	71	37	28	42
17	42	47	38	35	34
18	54	54	39	33	41
19	36	42	40	39	57
20	42	53	41	62	88
21	57	73	42	21	36

${{cV}}_{{{{O}}_{2} }}$  oxygen consumption by reverse Fick method, ${{mV}}_{{{{O}}_{2} }}$  oxygen consumption by indirect calorimetry

**Fig. 1:**
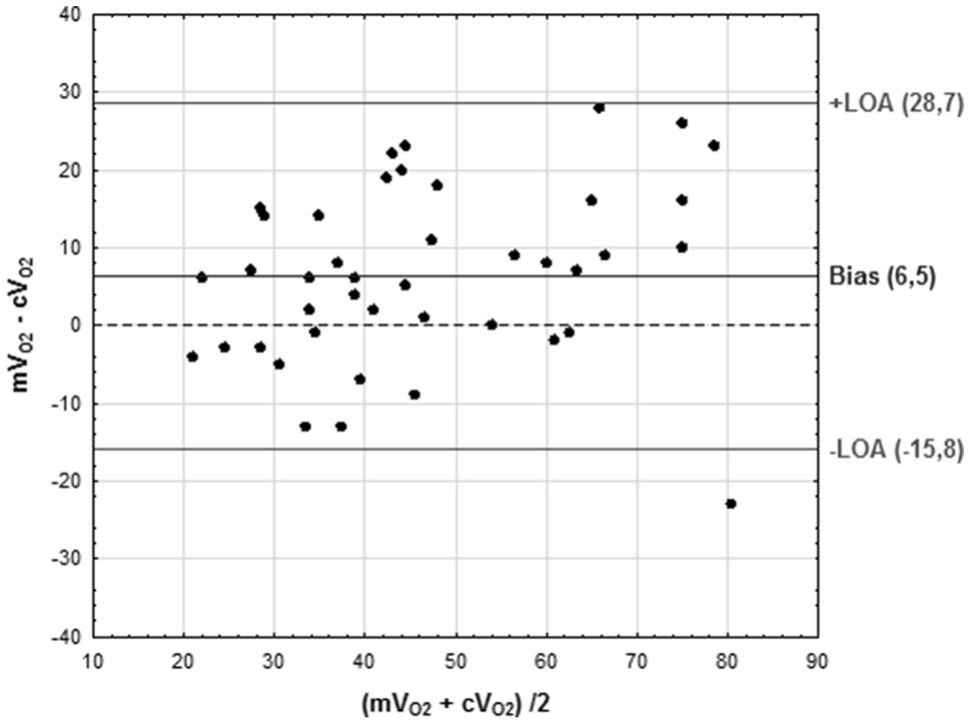
Bland–Altman plot comparing oxygen consumption with reverse Fick and (${\text{cV}}_{{{\text{O}}_{2} }}$) and indirect calorimetry (${\text{mV}}_{{{\text{O}}_{2} }}$)

The mean (SD) ${\text{cV}}_{{{\text{O}}_{2} }}$ was 43.5 (16.2) ml/min and ${\text{mV}}_{{{\text{O}}_{2} }}$ 49.9 (18.8) ml/min (*p* < 0.0001). Levene ‘s test verified normal distribution within the samples (*p* > 0.05) and Shapiro–Wilks test for the difference between methods $({\text{cV}}_{{{\text{O}}_{2} }} - {\text{mV}}_{{{\text{O}}_{2} }} )$ also indicated normal distribution (*p* = 0.92). Bias between ${\text{mV}}_{{{\text{O}}_{2} }}$ and ${\text{cV}}_{{{\text{O}}_{2} }}$ was 6.5 (11.3) ml/min (LOA of − 15.7 and 28.7 ml/min) corresponding to an overestimation by ${\text{mV}}_{{{\text{O}}_{2} }}$ of 14.7% (Fig. 1). The percentage error was 47.7%.

## Discussion

We found that the oxygen consumption measured by indirect calorimetry overestimated oxygen consumption calculated by the reverse Fick method by 14.7% in this cohort of young children after corrective heart surgery. The result from this study is consistent with findings in previous studies in adult patients where the overestimation has ranged between + 13 to + 30% [7, 8]. This difference has been explained in part by lung tissue oxygen consumption, but systematic errors and flaws in measurements (e.g. circuit leaks, calibration problems, possible oxygen consumption in blood gas samples at higher temperatures, lower oxygen uptake during anaesthesia and hyperdynamic response after cardiopulmonary bypass) may have influenced the results [23, 24]. A bias of this magnitude corresponds to a significant overestimation in CO of 0.19 l/min (from mean cardiac output of 1.28 l/min) in our cohort. As the direct Fick method is dependent on indirect calorimetry for estimation of cardiac output, this study indicates that this method is impaired by a considerable measurement error. Method comparison studies based on a reference technique already affected by this level of inaccuracy could unjustifily disqualify promising new alternatives for cardiac output measurements. This is especially true and extremly important in method comparison studies in children where the margin for error is less [25–27].

Statistical standards have evolved in the last few decades. In the beginning of cardiac output comparison studies, simple linear correlation coefficient analysis was used to compare two methods, but has since changed to Bland–Altman analysis [28]. Bland–Altman analysis is now required in all cardiac output comparison studies and a percentage error of less than 30% has been recommended between different methods in adult studies to be considered interchangeable [22]. Cecconi also highlighted another important fundamental demand in his review article regarding method comparison studies [29]. He insisted that the precision of the reference method be known and reported as it is a critical factor in differentiating between different methods. The precision of the direct Fick method is almost never available or reported as the sample of the respiratory gases has to be done over a certain time period and usually done together with only a single set of arterial and mixed venous blood gases for the cardiac output calculation. These limitations regarding precision were already known and addressed in the 1960s when direct Fick method was emerging as a new alternative for estimating of cardiac output [4]. In a recent systematic review and meta-analysis on accuracy and precision of minimally invasive cardiac output monitors in children, the direct Fick method was used as a reference method in 3 out of 20 studies [30]. In none of these studies was the precision of direct Fick method reported and, therefore, it was only possible to estimate if the tested methods were interchangeble. However, it was not known whether it was the new tested technique or the reference technique that contributed to the inaccuracy or the percentage error.

The direct Fick method is impractical in children as it is highly invasive and limited to general anesthesia in the catheterization lab. Although, the direct Fick method can be used to determine cardiac output in an individual child, it has to be kept in mind that systemic oxygen consumption is affected by metabolic needs and general anesthesia. Direct Fick method assumes equilibration between the measured pulmonary oxygen uptake and systemic oxygen consumption, which may be inaccurate during critical illness, high pulmonary oxygen consumption, and uncoupling between oxygen delivery and oxygen consumption. In addition, most metabolic evaluations relying on oxygen uptake measurements become difficult to interpret when higher fractions of inspiratory oxygen are needed.

## Conclusions

Direct Fick method is sensitive to minor changes or errors in saturation and oxygen consumption that can result in false cardiac output calculations. Indirect calorimetry seems to overestimate oxygen consumption in young children. Caution is advised when using the direct Fick method as a reference method in cardiac output method comparison studies in young children.

## Electronic supplementary material

Below is the link to the electronic supplementary material.

Supplementary file1 (XLSX 13 kb)
